# Clinical and Genomic Features of Androgen Indifferent Prostate Cancer

**DOI:** 10.3390/ijms26020679

**Published:** 2025-01-15

**Authors:** Jack Masur, Aakrosh Ratan, Krzysztof Wierbilowicz, Adanma Ayanambakkam, Michelle L. Churchman, Laura S. Graham, George Daniel Grass, Sumati Gupta, Sean Q. Kern, Jennifer King, Zin Myint, Robert J. Rounbehler, Bodour Salhia, Eric A. Singer, Yousef Zakharia, Bryce M. Paschal, Paul V. Viscuse

**Affiliations:** 1 Division of Hematology/Oncology, University of Virginia, Charlottesville, VA 22903, USA; nfm6jm@uvahealth.org; 2 Center for Public Health Genomics, University of Virginia, Charlottesville, VA 22903, USA; ar7jq@virginia.edu; 3 Department of Biochemistry and Molecular Genetics, University of Virginia School of Medicine, Charlottesville, VA 22903, USA; krzysztof1wierbilowicz@gmail.com (K.W.); bmp2h@virginia.edu (B.M.P.); 4 Stephenson Cancer Center, University of Oklahoma Health Sciences Center, Oklahoma City, OK 73104, USA; adanma-ayanambakkam@ouhsc.edu; 5 Aster Insights, Hudson, FL 34667, USA; michelle.churchman@asterinsights.com (M.L.C.); rob.rounbehler@asterinsights.com (R.J.R.); 6 Department of Medicine, Division of Medical Oncology, University of Colorado Cancer Center Anschutz Medical Campus, Aurora, CO 80045, USA; laura.graham@cuanschutz.edu; 7 H. Lee Moffitt Cancer Center, Department of Radiation Oncology, Tampa, FL 33612, USA; daniel.grass@moffitt.org; 8 Huntsman Cancer Institute, University of Utah, Salt Lake City, UT 84103, USA; sumati.gupta@hci.utah.edu; 9 Murtha Cancer Center, Uniformed Services University, Bethesda, MD 20814, USA; sean.q.kern.mil@health.mil; 10 Department of Medicine, Division of Hematology and Medical Oncology, Indiana University Health Melvin and Bren Simon Comprehensive Cancer Center, Indianapolis, IN 46202, USA; jmstrass@iu.edu; 11 Division of Medical Oncology, Department of Internal Medicine, Markey Cancer Center, University of Kentucky, Lexington, KY 40546, USA; zin.myint@uky.edu; 12 Norris Comprehensive Cancer Center, Keck School of Medicine, University of Southern California, Los Angeles, CA 90033, USA; salhia@usc.edu; 13 Division of Urologic Oncology, The Ohio State University Comprehensive Cancer Center, Columbus, OH 43210, USA; eric.singer@osumc.edu; 14 Holden Comprehensive Cancer Center, University of Iowa, Iowa City, IA 52242, USA; yousef-zakharia@uiowa.edu

**Keywords:** prostate cancer, androgen-indifferent prostate cancer, molecular biology, genomics, biomarkers, therapeutic targets

## Abstract

Androgen-indifferent prostate cancer (AIPC) is increasingly common and particularly lethal. Data describing these tumors are sparse, and AIPC remains a poorly understood malignancy. Utilizing the Oncology Research Information Exchange Network (ORIEN) database, we enriched for tumors with features of AIPC using previously described characteristics. Our AIPC cohort included three subgroups: aggressive variant prostate cancer (AVPC), neuroendocrine PC (NEPC), and double-negative PC (DNPC). Of 1496 total PC patients available for analysis, we identified 323 (22%) as MCRPC. Of those, 39 (12%) met AIPC criteria (17 AVPC, 13 NEPC, 9 DNPC) and 284 (88%) were non-AIPC. Forty-three percent of AIPC patients had de novo metastatic disease vs. 15% for non-AIPC (*p* = 0.003). Homologous recombination deficiency (HRD) and tumor mutational burden (TMB) did not differ between cohorts, but microsatellite instability scores (MSI) were significantly higher in AIPC (*p* = 0.019). Using Gene Set Enrichment Analysis (GSEA), we found that genes defining response to androgens and genes involved in oxidative phosphorylation were the most downregulated, whereas genes involved in epithelial–mesenchymal transition (EMT) and immune signaling were significantly upregulated in AIPC vs. non-AIPC. Our study demonstrates the potential for predefined criteria that aim to enrich for AIPC and suggests opportunities for therapeutic investigation.

## 1. Introduction

Prostate carcinogenesis is primarily driven by androgen receptor (AR) signaling. While androgen deprivation therapy (ADT) remains the mainstay of systemic treatment, the addition of AR signaling inhibitors (ARSIs) provides substantial improvement in overall survival and quality of life for many men with advanced prostate cancer [1,2,3,4,5,6,7]. However, resistance invariably develops, resulting in castrate-resistant prostate cancer (CRPC). While CRPC often retains reliance on AR signaling [8], approximately 20–30% of these resistant tumors represent androgen-indifferent prostate cancer (AIPC) [9,10,11,12]. AIPC is a distinct clinical and biological subset of prostate cancer with a particularly virulent course. Non-AR-targeted therapies, to date, have only led to incremental gains in survival outcomes [13,14,15,16].

Broadly, AIPC includes three subtypes that have various areas of overlap: neuroendocrine PC (NEPC), double-negative PC (DNPC), and aggressive variant PC (AVPC) [12]. NEPC refers to tumors with small-cell morphology that rarely presents as pure small-cell carcinoma and is often admixed with adenocarcinoma morphology [17]. NEPC can exist in primary untreated disease, but more commonly presents as treatment-emergent NEPC (t-NEPC) that arises following treatment with potent AR-targeted therapies [18]. DNPC refers to tumors that are AR-Null (do not express AR or PSA) and NE-Null (do not express synaptophysin). It has been hypothesized that these tumors represent an intermediate stage between AR-driven adenocarcinoma and NEPC following treatment with ARSIs [19]. The third subtype of AIPC is AVPC, which shares the clinical features and virulence of NEPC but includes those tumors that retain adenocarcinoma morphology [12]. In addition to clinical criteria, AVPC has also been defined by a molecular signature consisting of alterations in two out of three tumor suppressors: p53, RB1, PTEN [20].

Despite progress in recent years, defining clinical and molecular characteristics that overcome prostate cancer heterogeneity and enrich for AIPC remains an unmet need [12]. The absence of a consensus definition of AIPC impedes the development and proper study of novel therapies that may specifically benefit this subset of patients.

The Oncology Research Information Exchange Network (ORIEN) is an alliance of 19 NCI-designated cancer centers that share prospectively maintained clinical and molecular data for thousands of patients with various malignancies. We leveraged this contemporary database to further characterize molecular and clinicopathologic features of AIPC to detect identifiable biomarkers and reveal vulnerabilities that can be exploited therapeutically. We hypothesize that tumors meeting the criteria for NEPC, DNPC, and/or AVPC have distinct molecular characteristics with diagnostic and therapeutic potential compared with metastatic CRPC (mCRPC) not meeting these criteria.

## 2. Results

### 2.1. Baseline Demographics

**Figure 1:**
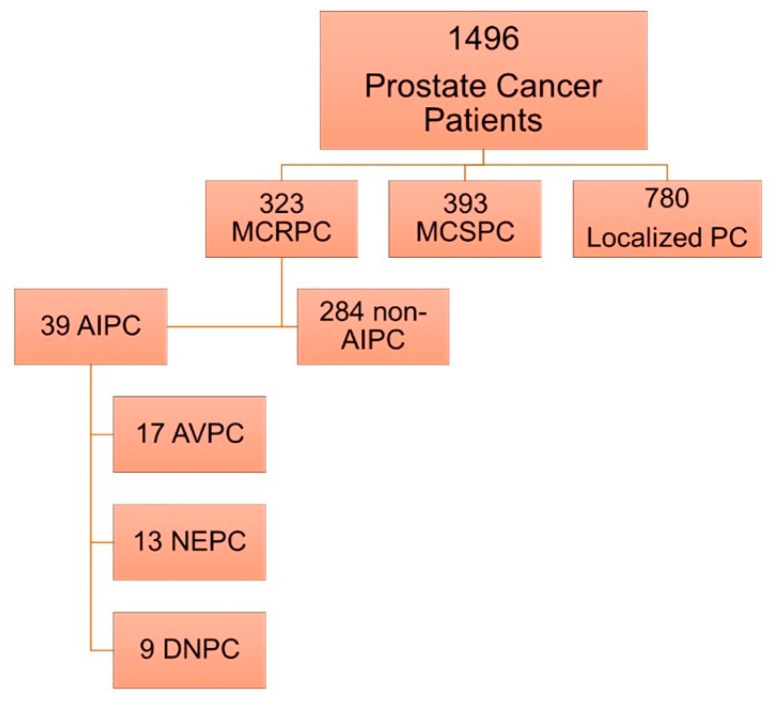
Results of AIPC enrichment query among all patients with prostate cancer in ORIEN database.

Data for 1496 patients diagnosed with prostate cancer were available for analysis in the ORIEN database. Of these, 323 (22%) were identified as mCRPC. Of the patients with mCRPC, 39 (12%) met our predefined AIPC criteria, and 284 (88%) were non-AIPC. Among the 39 AIPC samples, 13 (33%) were NEPC, 17 (44%) AVPC, and 9 (23%) DNPC (Figure 1). Of note, no patients had small-cell morphology reported on biopsy.

The median age at diagnosis for AIPC was 62 years, and 85% were White, compared with 62 years and 87% for non-AIPC. Forty-three percent of AIPC patients had de novo metastatic disease vs. 15% for non-AIPC (*p* = 0.003). The majority of AIPC samples were collected from the prostate gland (82%); other sites of collection for AIPC samples included lymph nodes (13%), bladder (2.5%), and CNS (2.5%). Non-AIPC samples were collected from the prostate gland (88%), lymph nodes (6%), bladder (2%), CNS (1.4%), bone (1.4%), liver (<1%), and esophagus (<1%) (Table 1). All specimens were taken at the time of initial prostate cancer diagnosis.

### 2.2. Clinical Outcomes

Among the 39 patients with AIPC, 10 (25.6%) were deceased at time of last follow-up, compared with 72 (25.4%) of the 284 patients with non-AIPC (Table 1). The median time from diagnosis to death or last follow-up was 68.4 months (17.0–328.7) for AIPC and 82.68 months (4.93–700.4) for non-AIPC (Table 1).

### 2.3. Differentially Expressed Genes in AIPC vs. Non-AIPC

**Figure 2:**
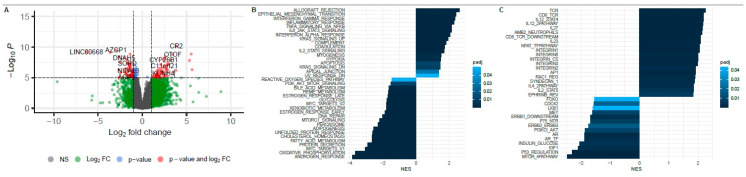
Differentially expressed genes in AIPC vs. non-AIPC: (**A**) Gene expression (mRNA) in AIPC vs. non-AIPC. (**B**) GSEA of hallmark performed on differentially expressed genes (AIPC vs. non-AIPC) detected by the Wilcoxon test (padj < 0.05). (**C**) Selected results of GSEA of Pathway Interaction Database (PID) performed on differentially expressed genes (AIPC vs. non-AIPC) detected by the Wilcoxon test (padj < 0.05).

We identified 295 genes with significantly higher mRNA expression in AIPC vs. non-AIPC samples (*p*-adjusted < 0.01) (Figure 2A).

Of the preferentially expressed genes in the AIPC cohort, the top ten protein-coding genes included *CR2*, *OTOF*, *KLHDC8A*, *CYP26B1*, *ELANE*, *C11orf21*, *TMEM132E*, *BLK*, *KRTAP10-8*, and *C12orf42* (Table 2).

In the non-AIPC cohort, 227 genes had significantly higher expression compared with AIPC (*p*-adjusted < 0.01) (Figure 2A). Of these, the top ten protein-coding genes included *AZGP1*, *ALOX15B*, *DCXR*, *NCAPD3*, *DNAH5*, *FASN*, *SLC39A6*, *SORD*, *STEAP2*, and *HIPK2* (Table 3).

**Figure 3:**
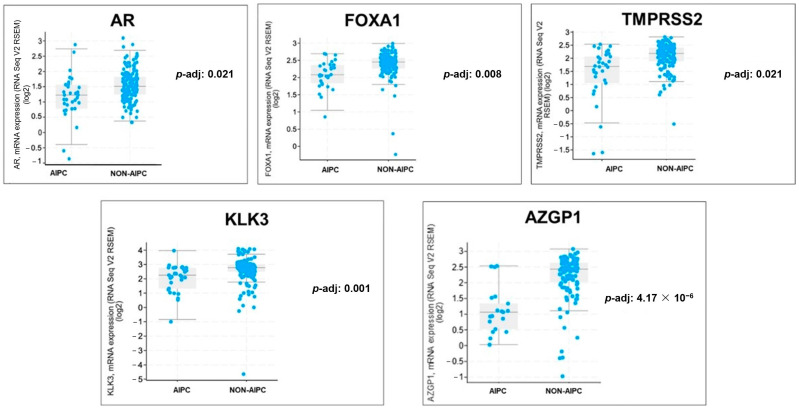
AR-associated gene expression (mRNA) in AIPC vs. Non-AIPC.

A large number of AR-associated genes had significantly lower expression in AIPC samples, including *AR*, *FOXA1*, *TMPRSS2*, *KLK3*, and *TMPRSS2* (Figure 3).

**Figure 4:**
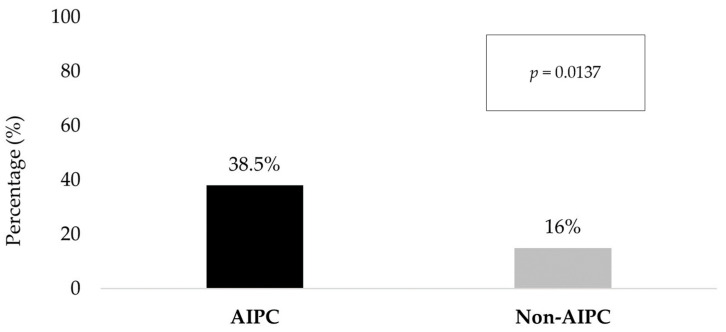
TMPRSS2-ERG gene fusions in AIPC vs. Non-AIPC.

Despite lower *TMPRSS2* expression in AIPC, TMPRSS2-ERG gene fusions were found in a significantly higher proportion of AIPC samples vs. non-AIPC (38.5% vs. 16%, *p* = 0.014) (Figure 4).

### 2.4. Gene Set Enrichment Analysis

Utilizing Gene Set Enrichment Analysis (GSEA), we identified several biological processes as summarized by hallmark gene sets that differed between AIPC and non-AIPC. Genes defining response to androgens and genes involved in oxidative phosphorylation were among the most downregulated in AIPC, whereas genes involved in epithelial–mesenchymal transition (EMT), interferon response, and inflammatory response were significantly upregulated in the three subgroups (AVPC, NEPC, DNPC) compared with unselected mCRPC samples (Figure 2B). Since we were interested in cellular signaling pathways, we also applied GSEA to the gene sets from the Pathway Interaction Database (PID). Genes involved in mTOR signaling, p53 regulation, IGF1 pathway, and AR pathway were most downregulated in AIPC samples, whereas genes involved in immune signaling through TCR signaling in CD4+ T-cells, IL12-mediated signaling events, and IL27-mediated signaling events were upregulated in AIPC samples (Figure 2C).

### 2.5. Immunosensitive Biomarkers

**Figure 5:**
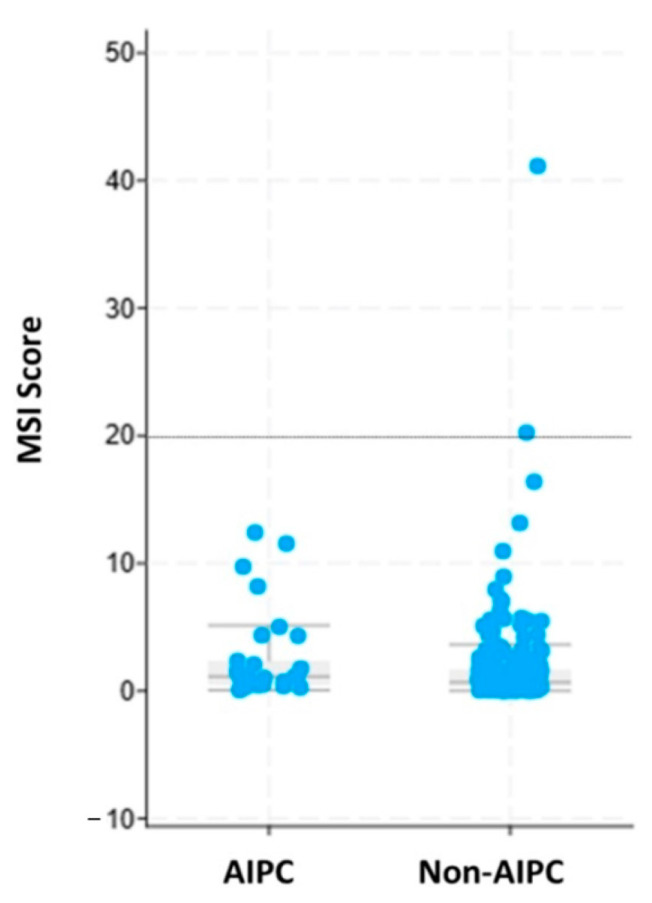
The cumulative distribution of MSI scores reported by ORIEN using MSIsensor2. The dotted line is the tool’s threshold to call a tumor MSI-positive.

Homologous recombination deficiency (HRD) did not differ significantly between cohorts, with a mean HRD score in AIPC of 5.85 and 6.46 in non-AIPC (*p* = 0.99). Tumor mutational burden (TMB) did not differ significantly between cohorts, with a mean TMB of 13.9 in AIPC and 9.05 in non-AIPC (*p* = 0.7). Microsatellite instability (MSI) score calculated using MSIsensor2 [21] was significantly higher in AIPC compared with non-AIPC (2.77 vs. 1.64, *p* = 0.019). Utilizing the recommended cutoff of 20% for MSIsensor2, zero of the AIPC samples and two (0.8%) non-AIPC samples were determined to be MSI-high (MSI-H) (Figure 5).

## 3. Discussion

The heterogeneity of metastatic prostate cancer and the overlap of previously defined subtypes of AIPC has presented a challenge in terms of effectively defining AIPC, which is further compounded by obstacles with tissue yield on biopsies at various times of progression. Our study utilized a contemporary database to try to enrich for and characterize AIPC among a broad population of patients with prostate cancer from 19 NCI-designated comprehensive cancer centers. We report on the clinical and genomic characteristics of these tumors to increase understanding of this poorly described subset of prostate cancer. We queried the ORIEN database for all patients with prostate cancer who subsequently developed mCRPC and found that 12% of tumor samples in this population enriched for AIPC using our prespecified criteria.

The term NEPC encompasses a broad spectrum of prostate cancers that have morphologic and IHC features consistent with neuroendocrine carcinoma [17]. While most NEPC tumors lack expression of AR and AR-regulated genes, this is not universally true [22]. Metastatic biopsy studies have identified mechanisms of lineage plasticity that downregulate the AR-driven luminal prostate adenocarcinoma and upregulate NEPC, but this process exists on a spectrum. The continuum of plasticity from an AR-driven state to AR independence makes it difficult to identify NEPC in the absence of tissue biopsy. In order to enrich for NEPC tumors in this study, we utilized the 70-gene signature integrated NEPC score that distinguishes NEPC from metastatic prostate adenocarcinoma [23]. Compared with mCRPC, NEPC often exhibits low AR expression and diminished AR signaling, and subsequently, there is decreased expression of AR target genes [12,17,19,24]. As such, the integrated NEPC score has been shown to be inversely correlated with AR signaling in mCRPC samples [23]. Our genomic analysis of primary untreated tumors in the ORIEN database also found the NEPC score to be inversely correlated with AR signaling. Looking specifically at AR-associated gene expression in our treatment-naïve primary prostate cancer samples, our AIPC cohort demonstrates significantly lower expression of *AR*, *FOXA1*, *TMPRSS2*, *KLK3*, *AZGP1*, and several other AR-associated genes when compared with the non-AIPC cohort. Pathway analysis via GSEA comparing our AIPC vs. non-AIPC cohorts also shows that genes defining response to androgens were the most significantly downregulated. This suggests that we are effectively enriching for androgen indifference with our prespecified criteria.

Spratt and colleagues found that the AR-low, treatment-naïve primary prostate tumors that they selected for were characterized by rapid development of recurrence or metastasis [25]. Similarly, we found evidence of more aggressive disease among our AIPC cohort compared with non-AIPC. Analyzing the available clinical data in ORIEN, we found that 43% of patients in the AIPC cohort presented with de novo metastatic disease compared with 15% in the non-AIPC cohort. This finding suggests that patients whose primary tumors enriched for AIPC via our methodology had more aggressive disease. Our GSEA analysis also suggests enrichment for aggressive disease among our AIPC cohort, as it was demonstrated that genes involved in EMT were significantly upregulated in the three subgroups (NEPC, DNPC, AVPC) compared with non-AIPC samples. The transcription factor *SPDEF*, which has been reported to suppress metastasis via inhibition of EMT in prostate cancer and is known to have low expression in NEPC compared with CRPC, also demonstrated lower mRNA expression in our AIPC cohort compared with the non-AIPC cohort [12]. There was no observed difference in overall survival outcomes between the two groups; however, this result is difficult to interpret due to the small sample size in the AIPC cohort and the likelihood of multiple confounding factors, including initial therapy, patient-specific characteristics including comorbidities, and subsequent treatments, among others.

While our transcriptomic analysis does not reveal direct therapeutic targets, there may be opportunities for therapeutic investigation. Platinum-based chemotherapy, such as carboplatin plus cabazitaxel, has been observed to have efficacy in tumors with the AVPC molecular signature [26]. Responses to platinum-based treatments are short-lived, however, and there is no clear guidance for additional therapy. Our GSEA analysis demonstrates that genes involved in immune signaling were significantly more upregulated in AIPC, suggestive of a potential role for immune-based treatments such as immune checkpoint inhibitors which have had disappointing results in all-comer mCRPC populations to date [27,28,29].

In our analysis, there was no difference in TMB or HRD between AIPC and non-AIPC patients. We did find a statistically significant difference in the MSI score between the two cohorts, with the mean score being higher in AIPC; however, it is important to note that none of the AIPC samples and just two (<1%) non-AIPC samples were above the accepted threshold to be considered MSI-high. Understanding the prevalence and application of TMB, HRD, and microsatellite instability in AIPC remains an open question. While immune checkpoint inhibitors are often used in the treatment of small-cell lung cancer and sometimes extrapolated to the treatment of NEPC, their efficacy has not yet been specifically described in AIPC.

Our GSEA analysis also demonstrates that genes involved in oxidative phosphorylation are significantly more downregulated in AIPC. Downregulation of genes associated with oxidative phosphorylation often leads to upregulation of angiogenesis-related genes via hypoxia-driven signaling, particularly through HIF-1α activation, as this adaptive response aims to restore oxygen and nutrient availability in the tumor microenvironment and enable continued tumor growth and progression.

Our analysis found that genes associated with angiogenesis were indeed downregulated; however, it did not pass the threshold for significance with an adjusted *p*-value of 0.065. It is possible that a subset of patients exhibit this signature and a larger sample size and/or an analysis of the heterogeneity of the cohort is warranted. Nevertheless, our GSEA findings suggest a possible role for treatments that target hypoxia-driven signaling and/or angiogenic signaling. The CONTACT-02 trial was a phase III study evaluating the combination of cabozantinib and atezolizumab versus a second novel hormonal therapy in patients with mCRPC who had progressed on a prior novel hormonal agent [30]. While the combination did not meet the primary endpoints of significant overall survival (OS) or progression-free survival (PFS) improvement in the overall cohort, subgroup analyses revealed a notable survival benefit in patients with liver metastases. With visceral metastasis as a clinical feature of AVPC, these findings may suggest a particular benefit with angiogenesis inhibitors and warrant further study in this subset of patients while highlighting the importance of patient selection.

Increased understanding of the genetic drivers of certain advanced prostate cancer subtypes has highlighted the importance of genetic testing to help guide tailored therapeutic approaches for patients. The presence of BRCA germline mutation, for instance, has been shown to influence not only disease incidence and survival outcomes but also the effectiveness of treatments [31]. Translational successes with PARP inhibitors for patients with advanced BRCA-mutated prostate cancer have led to improved clinical outcomes for this subset of patients and several recent FDA approvals [32,33,34]. While BRCA1/BRCA2 mutations impair DNA repair through homologous recombination leading to genomic instability, AIPC is characterized by aggressive disease that often arises after androgen deprivation therapy fails, driven by alternative genetic alterations or pathways. The PROREPAIR-B study showed that patients with metastatic prostate cancer and germline BRCA2 mutations exhibited a shorter time to developing castration resistance compared with non-carriers, suggesting that tumors in BRCA2 mutation carriers may be less reliant on AR signaling pathways. Thus, the understanding of certain genetic drivers of advanced prostate cancer, such as BRCA mutations, may provide a framework for exploring overlaps between genetic instability and the progression to androgen independence in prostate cancer. This may inform strategies to manage AIPC using precision medicine principles, leveraging insights from BRCA-driven pathways and other genetically tailored therapies.

Our study had several limitations, the first of which was that it was performed in a retrospective manner and thus may have unknown sources of bias. Despite having patients from multiple NCI-designated comprehensive cancer centers, the overall population size was small which limits interpretation of the prevalence of and findings for the AIPC cohort. Our findings must also be viewed within the inherent limitations of a database analysis, including missing or incomplete clinical data. The availability of more descriptive patient characteristics, clinical data, treatment records, progression events, and patient outcomes would strengthen this investigation.

A particular limitation of our study is the timepoint at which the tumor was obtained in relation to the patients’ overall clinical course. Tissue samples available for analysis via the ORIEN database were predominantly obtained at the time of original prostate cancer diagnosis. Previous studies suggest that most AR-independent NEPC emerges via lineage plasticity, in which selective pressures from intensive AR suppression drive histologic transformation from prostate adenocarcinoma to small-cell NEPC [19,24,35]. In the era of widespread potent ARSI use, AIPC prevalence is increasing. This is highlighted by an autopsy study of men with mCRPC, which shows that the proportion of patients with AR-negative tumors was 11.6% in the pre-ARSI era (1998–2011) and 36.7% in the modern ARSI era (2012–2016) [19]. Despite a growing understanding of the mechanisms that drive the development of androgen resistance, widespread characterization of these tumors is sparse. While we selected for patients who were known to subsequently develop mCRPC, the analyzed tissue samples were taken at the time of diagnosis and thus our AIPC cohort does not capture t-NEPC and likely underestimates the true incidence of AIPC within the ORIEN database.

Although most NEPC features may not typically be detected early in the disease course, it is likely that early transcriptomic changes associated with AR independence exist [25,36]. A 2019 study by Spratt and colleagues analyzed genome-wide expression profiles of treatment-naïve primary prostate cancer tissue samples and identified a low AR-active subclass in about 10% of tumors [25]. While our study attempts to more broadly enrich for AIPC based on predefined criteria for NEPC, DNPC, and AVPC as opposed to selecting tumors solely based on AR expression, we found a similar prevalence of androgen-indifferent tumors (12%) among primary prostate cancer samples analyzed in the ORIEN database. Though most molecular alterations leading to androgen indifference are thought to be acquired later in a patient’s disease and treatment course, there may also be certain molecular features present at initial diagnosis that contribute to the development of AR independence [22].

## 4. Materials and Methods

Utilizing the ORIEN network, we queried all patients with a prostate cancer diagnosis sequenced in the ORIEN database. We then selected for the patients diagnosed with mCRPC. We defined mCRPC as a patient who (1) received at least one of a predefined list of AR-targeted prostate cancer medications inducing castration and (2) had a progression event after the medication was administered. To discriminate between non-metastatic castrate-resistant prostate cancer (nmCRPC) and mCRPC, we only included patients with evidence of metastasis on imaging. Patients with a progression event included those with new metastases seen on imaging and/or worsening metastatic volume detected on imaging. Precise measurements or descriptions of the volume of disease among individual patients were not available in the database.

We analyzed clinical data and gene expression profiles for patients with mCRPC and compared samples that enriched for AIPC (“AIPC”) with those that did not enrich for AIPC (“non-AIPC”). We attempted to enrich for AIPC using previously described characteristics for NEPC, DNPC, and AVPC. In this study, NEPC was defined as samples with either small-cell morphology on biopsy or samples with an integrated NEPC signature score ≥ 0.25 [23]. NEPC did not include adenocarcinoma with positive chromogranin/synaptophysin staining by IHC alone, as this has not been shown to correlate with biology [37]. DNPC was defined as samples not selected as NEPC per the aforementioned criteria and that were AR low, defined as an overlap of the bottom quartile AR expression and AR signaling score, utilizing a previously defined RNA-based signature [38]. We did not use the traditional IHC definition for DNPC due to the limitations of the database [19]. AVPC was defined as having genomic alterations in at least two of *TP53*, *RB1*, *PTEN* [38].

Clinical analysis was performed using the Wilcoxon rank sum test or Fisher’s exact test. For gene expression analysis, we included all formalin-fixed samples from primary tumors and used DESeq2 [39] to identify differentially expressed genes comparing AIPC samples to non-AIPC samples after accounting for the RNA batch. Gene Set Enrichment Analysis (GSEA) [40] of hallmark and Pathway Interaction Database (PID) gene sets in MSigDB was performed using the fgsea R package version 4.4.1 after ranking the genes based on the Wald statistic.

## 5. Conclusions

Our study demonstrates the potential for predefined criteria that aim to enrich for AIPC, supported by the higher rate of de novo metastasis, as well as the downregulated androgen response and upregulated EMT pathways in the AIPC cohort. Upregulated immune signaling, higher MSI, and lower expression of oxidative phosphorylation pathways suggest opportunities for therapeutic investigation. Future directions include validation of the predefined criteria using other databases, leverage of tissue-rich prospective studies to further characterize these tumors beyond clinical features, and design of in vitro and in vivo studies to explore potential vulnerabilities informed by such analyses.

## Figures and Tables

**Table 1:** Baseline characteristics and outcomes of patients with AIPC vs. Non-AIPC.

	AIPC	Non-AIPC	
Median age at diagnosis (IQR)	62 years (56–68)	62 years (57–67)	
Race			
Caucasian	85%	87%	
African American	10%	9%	
Asian	2.5%	2%	
Unknown	2.5%	3%	
Metastatic disease at diagnosis	44%	15%	*p* = 0.003
Specimen site of collection			
Prostate gland	82%	88%	
Lymph node	13%	6%	
Bladder	2.5%	2%	
CNS	2.5%	1.4%	
Bone	0%	1.4%	
Liver	0%	<1%	
Esophagus	0%	<1%	
Median time from diagnosis to death or last follow up (range)	68.4 months (17.0–328.7 months)	82.7 months (4.93–700.4 months)	
Clinical Outcome at last follow up			
Deceased	25.6%	25.4%	
Alive	74.6%	74.4%	

**Table 2:** Top 10 genes preferentially expressed (mRNA) in AIPC vs. Non-AIPC.

Gene	*p*-adj
*CR2*	1.83 × 10^−6^
*OTOF*	5.93 × 10^−6^
*KLHDC8A*	1.72 × 10^−5^
*CYP26B1*	2.39 × 10^−5^
*ELANE*	7.86 × 10^−5^
*C11orf21*	1.07 × 10^−4^
*TMEM132E*	1.94 × 10^−4^
*BLK*	3.00 × 10^−4^
*KRTAP10-8*	3.09 × 10^−4^
*C12orf42*	3.09 × 10^−4^

**Table 3:** Top 10 genes preferentially expressed (mRNA) in Non-AIPC vs. AIPC.

Gene	*p*-adj
*AZGP1*	4.17 × 10^−6^
*ALOX15B*	4.88 × 10^−6^
*DCXR*	5.93 × 10^−6^
*NCAPD3*	1.09 × 10^−5^
*DNAH5*	1.51 × 10^−5^
*FASN*	2.39 × 10^−5^
*SLC39A6*	5.98 × 10^−5^
*SORD*	6.48 × 10^−5^
*STEAP2*	7.86 × 10^−5^
*HIPK2*	8.01× 10^−5^

## Data Availability

All data generated and/or analyzed in this study are either available on the Gene Expression Omnibus or are available from the corresponding author upon reasonable request.
